# The U-Rich Untranslated Region of the Hepatitis E Virus Induces Differential Type I and Type III Interferon Responses in a Host Cell-Dependent Manner

**DOI:** 10.1128/mBio.03103-19

**Published:** 2020-01-14

**Authors:** Harini Sooryanarain, Connie L. Heffron, Xiang-Jin Meng

**Affiliations:** a Department of Biomedical Sciences and Pathobiology, Virginia-Maryland College of Veterinary Medicine, Virginia Polytechnic Institute and State University, Blacksburg, Virginia, USA; Icahn School of Medicine at Mount Sinai

**Keywords:** hepatitis E virus (HEV), U-rich region RNA PAMPs, retinoic acid-inducible gene I (RIG-I), type I interferon (IFN), type-III IFN

## Abstract

Hepatitis E virus (HEV), a single-strand positive-sense RNA virus, is an understudied but important human pathogen. The virus can establish infection at a number of host tissues, including the small intestine and liver, causing acute and chronic hepatitis E as well as certain neurological disorders. The retinoic acid-inducible gene I (RIG-I) pathway is essential to induce the interferon (IFN) response during HEV infection. However, the pathogen-associated motif patterns (PAMPs) in the HEV genome that are recognized by RIG-I remain unknown. In this study, we first identified that HEV RNA PAMPs derived from the 3′ untranslated region (UTR) of the HEV genome induced higher levels of IFN mRNA, interferon regulatory factor-3 (IRF3) phosphorylation, and nuclear translocation than the 5′ UTR of HEV. We revealed that the U-rich region in the 3′ UTR of the HEV genome acts as a potent RIG-I PAMP, while the presence of poly(A) tail in the 3′ UTR further increases the potency. We further demonstrated that HEV UTR PAMPs induce differential type I and type III IFN responses in a cell type-dependent fashion. Predominant type III IFN response was observed in the liver tissues of pigs experimentally infected with HEV as well as in HEV RNA PAMP-induced human hepatocytes *in vitro*. In contrast, HEV RNA PAMPs induced a predominant type I IFN response in swine enterocytes. Taken together, the results from this study indicated that the IFN response during HEV infection depends both on viral RNA motifs and host target cell types. The results have important implications in understanding the mechanism of HEV pathogenesis.

## Abstract (precis)

Hepatitis E virus (HEV) is an important human pathogen causing both acute and chronic viral hepatitis E infection. Currently, the mechanisms of HEV replication and pathogenesis remain poorly understood. The innate immune response acts as the first line of defense during viral infection. The retinoic acid-inducible gene I (RIG-I)-mediated interferon (IFN) response has been implicated in establishing antiviral response during HEV infection, although the HEV RNA motifs that are recognized by RIG-I are unknown. This study identified that the U-rich region in the 3′ untranslated region (UTR) of the HEV genome acts as a potent RIG-I agonist compared to the HEV 5′ UTR. We further revealed that the HEV RNA pathogen-associated motif patterns (PAMPs) induced a differential IFN response in a cell type-dependent manner: a predominantly type III IFN response in hepatocytes, and a predominantly type I IFN response in enterocytes. These data demonstrate the complexity by which both host and viral factors influence the IFN response during HEV infection.

## INTRODUCTION

Hepatitis E virus (HEV), a single-strand positive-sense RNA virus, belongs to the family *Hepeviridae* consisting of two distinct genera, *Orthohepevirus* and *Piscihepevirus*. The species *Orthohepevirus* A includes virus strains that infect humans and is subclassified into at least eight different genotypes (1). Genotypes 1 to 4 HEVs are of significant human health importance (2). Genotypes 1 and 2 HEVs infect only humans, usually establish acute infection associated with large explosive outbreaks, and can cause an increased mortality in infected pregnant women (3). Genotypes 3 and 4 HEVs are zoonotic in nature, infect humans and several other animal species, including pigs (4), can establish chronic infection in immunocompromised patients (5), and can cause neurological diseases in some cases (6). Annually, it is estimated that ∼20 million people are infected by HEV, and approximately 44,000 die of HEV-related diseases (7). HEV is usually transmitted through the fecal-oral route via contaminated water or food, with a primary site of virus replication at the small intestine (8), before establishing an infection at the target organ—the liver. Currently, the mechanism of HEV immunopathogenesis remains elusive. Investigation of the immune responses at the primary site of HEV infection as well as at the target organ would provide us with a better understanding of HEV pathogenesis.

The innate immune response forms the first line of defense against viral infections, including HEV. Retinoic acid-inducible gene I (RIG-I) senses pathogen-associated motif patterns (PAMPs) in viral RNAs to induce antiviral innate immune responses. RIG-I belongs to a family of DExD/H helicases, which have both nucleic acid-binding properties and ATP hydrolysis activity (9). RIG-I recognition of the viral RNA PAMPs depends on the PAMP motif length, the 5′ end modification (capped or presence of free phospho group), and nucleotide composition (9). The binding of RIG-I and viral RNA motifs signals the downstream transcription factor activation, which subsequently induces type I and/or III interferon (IFN) expression to establish an antiviral state (10). The RIG-I pathway has been shown to play an important role during HEV infection (11, 12). However, the HEV RNA motifs that are recognized by RIG-I remain unknown.

The genomic RNA of HEV is ∼7.2 kb in size, comprising a 5′ untranslated region (UTR), open reading frame (ORF) 1 encoding nonstructural proteins, ORF2 encoding capsid protein, ORF3 encoding membrane ion channel-like protein (13), and a 3′ UTR (14). ORF2 and ORF3 are expressed as subgenomic RNA and partially overlap, but neither overlap ORF1 (14). In addition to the 5′ UTR and 3′ UTR, the HEV genome also contains a stem-loop structure located in the junction region between the 3′ end of ORF1 and the 5′ end of the ORF3-ORF2 (15, 16). This stem-loop structure in the junction region is crucial for subgenomic RNA expression and viral replication (16, 17). Viral UTRs, in addition to playing an important role in viral replication, also act as PAMPs and are recognized by host pattern recognition receptors (PRRs) such as RIG-I (9).

In this study, we aimed to investigate the RIG-I activation potential of HEV RNA UTRs. Since HEV establishes primary infection in the small intestine before reaching its target organ (the liver), we also determined and compared the IFN responses induced by HEV PAMPs in hepatocytes as well as enterocytes. The results from this study revealed that the U-rich region of HEV UTRs induces a differential type I and type III IFN response in a host cell type-dependent manner.

## RESULTS

### Differential induction of type I (α) and type III (λ1) IFN mRNA expression levels in liver tissues of conventional and gnotobiotic pigs experimentally infected with a genotype 3 human HEV.

**FIG 1:**
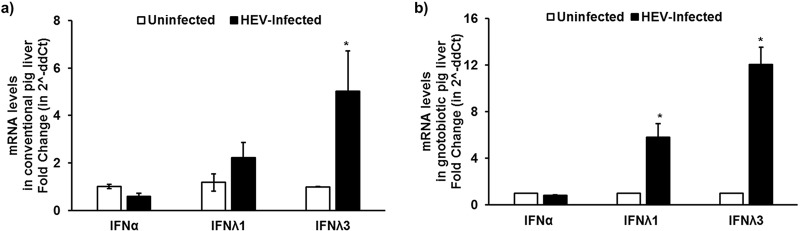
Differential induction of type I (α) and type III (λ1/3) IFN mRNA expression levels in liver tissues of conventional and gnotobiotic pigs experimentally infected with a genotype 3 human HEV. Type I (IFN-α) and type III (IFN-λ1/3) IFN mRNA expression levels in uninfected or HEV-infected conventional (*N* = 2 uninfected, 4 infected) (a) or gnotobiotic (*N* = 5 uninfected, 6 infected) (b) pigs at 4 weeks postinfection (wpi) were quantified using gene-specific RT-qPCR. Swine RSP32 was used as a housekeeping control. Fold change was calculated using the threshold cycle (2^−ΔΔ^*^CT^*) method. *, *P* < 0.05 versus uninfected pigs. The data represent means ± standard errors of the means (SEMs).

By using the liver tissues of conventional (18) and gnotobiotic (19) pigs experimentally infected with a genotype 3 human HEV from prior studies, we quantified the mRNA expression levels of type I (alpha interferon [IFN-α]) and type III IFN (IFN-λ1 and -λ3) in infected pig livers at 4 weeks postinfection (wpi) using gene-specific reverse transcription-quantitative PCR (RT-qPCR). Our results showed that there were significantly increased type III IFN (λ1 and λ3) mRNA expression levels in the liver tissues of HEV-infected conventional pigs (Fig. 1a) (*N* = 4) and gnotobiotic pigs (Fig. 1b) (*N* = 6) compared to that in the uninfected control pigs (*N* = 5). However, there was no difference in the type I IFN-α mRNA expression levels (Fig. 1a and b). The results revealed a differential induction of types I and type III IFN mRNA levels in liver tissues of HEV-infected pigs. This finding was in corroboration with previous published studies, further demonstrating that HEV infection leads to a predominant type III IFN response in liver cells (20). However, the HEV RNA motifs that are recognized by RIG-I as viral PAMPs remain unknown. Therefore, we conducted further experiments in this study to identify the HEV RNA PAMPs.

### The 3′ UTR of the HEV genome induced higher levels of IFN mRNAs in Huh7-S10-3 liver cells via the RIG-I pathway.

**FIG 2:**
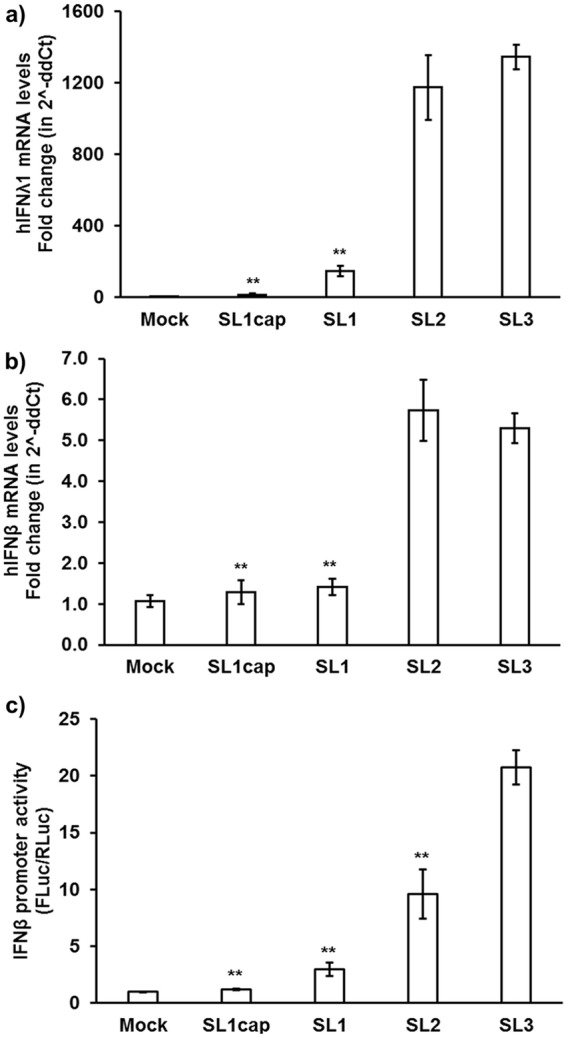
HEV 3′ UTR induced higher levels of IFN mRNAs in Huh7-S10-3 liver cells. Type III IFN (hIFN-λ1) (a) and type I IFN (hIFN-β) (b) mRNA levels in plain Huh7-S10-3 cells stimulated by different HEV RNA PAMPs (SL1cap, SL1, SL2, or SL3) at 18 h poststimulation were quantified using gene-specific qPCR. The fold change was calculated compared to unstimulated cells (mock), using the 2^−ΔΔ^*^CT^* method. RPS18 was used as a housekeeping control. (c) IFN-β promoter activity in HEV RNA PAMP-stimulated Huh7-S10-3 cells and unstimulated (mock) cells. Human IFN-β promoter firefly luciferase was used as a reporter plasmid, and TK-*Renilla* luciferase was used as a control vector. The IFN-β promoter activity was calculated by determining the ratio of firefly luciferase (FLuc)/*Renilla* luciferase (RLuc) levels as measured by Dual-Glo kit. **, *P* ≤ 0.01 versus HEV SL3 using paired Student *t* test. The data represent means ± SEMs of results from three independent experiments.

To identify the HEV RNA motifs that are recognized as PAMPs by RIG-I, Huh7-S10-3 liver cells were stimulated with various HEV RNA PAMPs, including HEV capped 5′ UTR (SL1-cap), 5′ UTR without cap (SL1), CRE-JR stem-loop (SL2), and 3′ UTR with poly(A) (SL3) RNAs. At 18 h poststimulation, the mRNA levels of IFN-λ1 and IFN-β were quantified using gene-specific RT-qPCR. We chose these regions of the viral genome to test as potential HEV RNA PAMPs, because RIG-I preferentially binds to short RNA of <300 bp (21) and these regions in HEV genomic/subgenomic RNA also contain UTRs. The results showed that stimulation of plain Huh7-S10-3 liver cells with the HEV SL3 (3′ UTR) led to a significant increase in IFN-β promoter activity and mRNA expression levels of IFN-λ1 (Fig. 2a) (*P* < 0.01) and IFN-β (Fig. 2b) (*P* < 0.01) compared to that with the HEV SL1 (5′ UTR). We also found that the presence of cap in SL1 led to a significant reduction in SL1 IFN-inducing capacity. The SL2-stimulated liver cells had similar levels of IFN mRNA expression, while the IFN-β promoter activation levels were lower than those of the SL3-stimulated cells.

**FIG 3:**
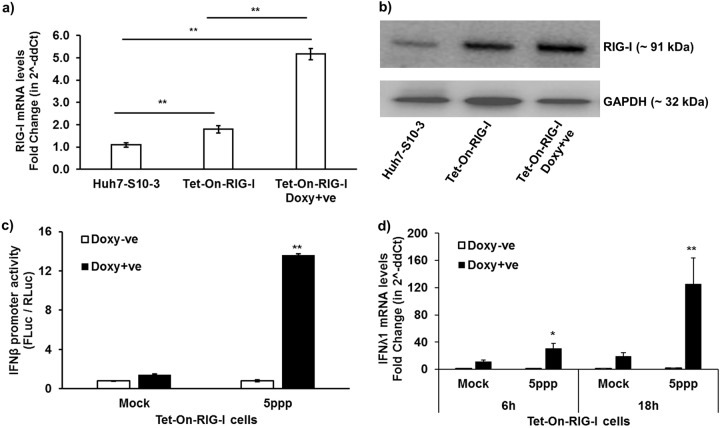
Establishment of Tet-On RIG-I Huh7-S10-3 liver cells. The Tet-On RIG-I Huh7-S10-3 cells were established by transducing human Huh7-S10-3 with pLIX402 lentivirus containing hRIG-I CDS under a doxycycline-inducible promoter. RIG-I mRNA levels (a) and RIG-I protein levels by Western blotting (b) in plain Huh7-S10-3 cells and Tet-On RIG-I cells in the presence or absence of doxycycline. The Tet-On RIG-I cells were stimulated with RIG-I agonist 5′ppp ligand (5ppp) or left unstimulated (mock), and cultured in the presence (Doxy+ve) or absence (Doxy-ve) of doxycycline (5 μg/ml). (c) IFN-β promoter activity at 12 h poststimulation of Tet-On RIG-I cells with 5′ppp or left unstimulated. IFN-β promoter activity was calculated by determining the ratio of firefly luciferase (FLuc)/*Renilla* luciferase (RLuc) levels as measured by Dual-Glo kit. (d) Type III IFN (IFN-λ1) mRNA expression levels in 5′ppp-stimulated Tet-On RIG-I cells at 6 h and 18 h poststimulation were quantified using gene-specific RT-qPCR. The fold change was calculated compared to unstimulated cells (mock) using the 2^−ΔΔ^*^CT^* method. RPS18 was used as a housekeeping control. *, *P* < 0.05; **, *P* < 0.01 versus mock. Data represent means ± SEMs of results from three independent experiments.

To further confirm the role of RIG-I in the HEV UTR-induced IFN response, we determined the IFN-λ1 mRNA and IFN-β promoter activity levels in Huh7-S10-3 cells overexpressing human RIG-I (hRIG-I) under a doxycycline-inducible promoter. The Huh7-S10-3 cells were transduced with the Tet-On human-RIG-I lentivirus to establish a Tet-On Huh7-S10-3-hRIG-I cell line (Fig. 3a and b). We found that stimulation of Tet-On Huh7-S10-3-hRIG-I cells with RIG-I agonist ligand 5′ triphosphate (5′ppp; Invivogen), in the presence of doxycycline, led to a significant increase in IFN-β promoter activity (Fig. 3c) and IFN-λ1 mRNA levels (Fig. 3d). We also observed that overexpression of RIG-I decreased HEV infectious titer (focus-forming units [FFU] per milliliter) and intracellular HEV RNA levels at 6 to 7 days postinfection (dpi). Infection of plain Huh7-S10-3 liver cells with the genotype 3 HEV Kernow P6 virus yielded 2.5 ± 0.5 log_10_ FFU/ml infectious virus and 10,270 ± 1,144 HEV RNA copies/ml, while overexpression of RIG-I reduced the virus titer by 10-fold, i.e., to 1.5 ± 0.5 log_10_ FFU/ml and 3,202 ± 569 HEV RNA copies/ml. These results indicate that the Tet-On Huh7-S10-3-hRIG-I cells overexpressed functional RIG-I.

**FIG 4:**
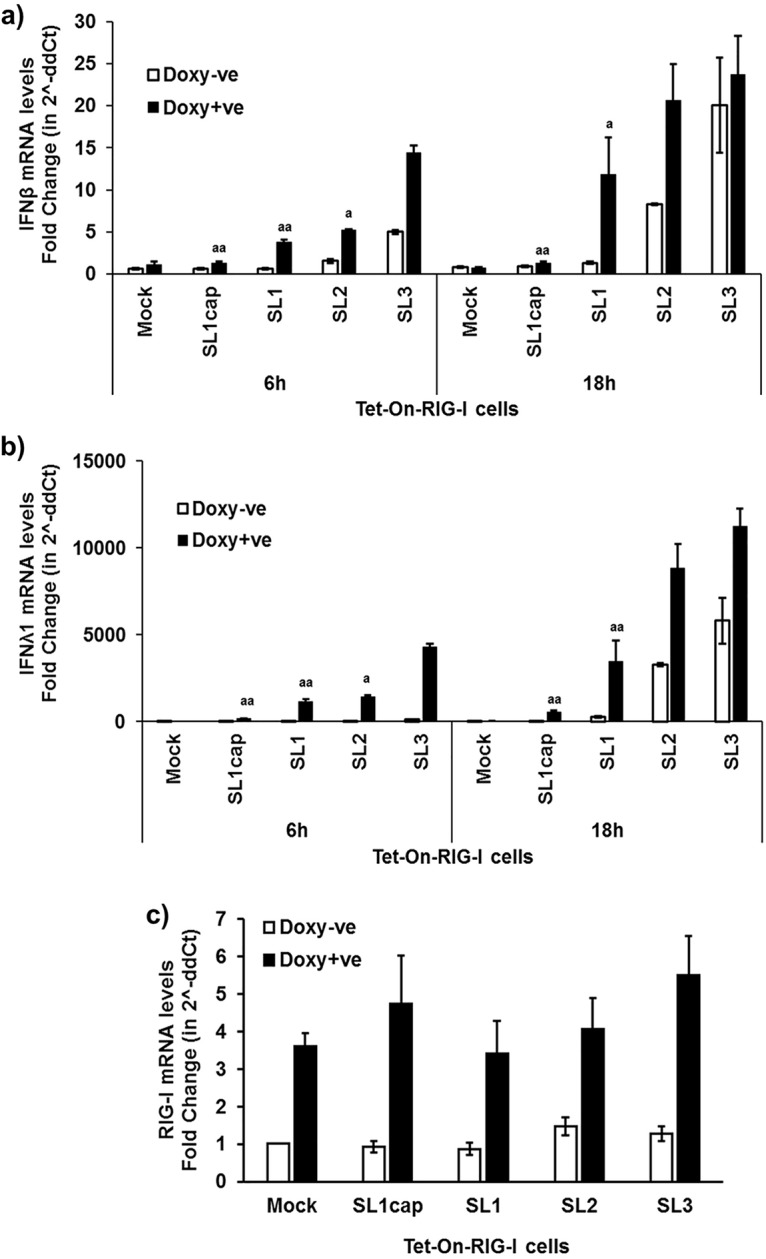
Type I and type III IFN mRNA expression levels in HEV RNA PAMP-stimulated Tet-On RIG-I Huh7-S10-3 liver cells. Type I IFN (hIFN-β) (a) and type III IFN (hIFN-λ1) (b) mRNA expression levels in HEV RNA PAMP (SL1cap, SL1, SL2, or SL3)-stimulated Tet-On RIG-I cells at 6 h and 18 h poststimulation were quantified using gene-specific RT-qPCR. (c) RIG-I mRNA levels at 18 h poststimulation. The fold change was calculated compared to the unstimulated cells (mock) using the 2^−ΔΔ^*^CT^* method. RPS18 was used as a housekeeping control. ^a^, *P* < 0.05; ^aa^, *P* < 0.01 versus HEV SL3-stimulated cells. Data represent means ± SEMs of results from two independent experiments.

To determine the IFN induction capacity by different HEV-RNA PAMPs, the Tet-On Huh7-S10-3-hRIG-I cells were stimulated with HEV SL1cap, SL1, SL2, and SL3 in the presence or absence of doxycycline. The results showed that doxycycline induced overexpression of RIG-I, leading to further enhanced IFN-λ1 mRNA expression levels in HEV RNA PAMP-stimulated cells compared to those in cells without doxycycline treatment. HEV SL3 induced significantly higher IFN-β (Fig. 4a) and IFN-λ1 (Fig. 4b) mRNA expression levels than the HEV SL1 at 6 h and 18 h poststimulation. We also observed that HEV SL3 induced higher levels of IFN-λ2 and IFN-λ3/2 mRNA than HEV SL1 at 18 h poststimulation, as seen in the case of IFN-λ1 mRNA levels (see Fig. S1 in the supplemental material). Type III IFN mRNA expression was more predominant than type I IFN mRNA expression in HEV PAMP-stimulated samples (Fig. 4), and this was consistent with our *in vivo* results using liver tissues of HEV-infected gnotobiotic and conventional pigs (Fig. 1). Since IFN-λ1, IFN-λ2, and IFN-λ3/2 mRNA levels in HEV PAMP-stimulated cells followed similar trends, we therefore used IFN-λ1 as a marker to monitor type III IFN levels in further *in vitro* experiments in this study.

10.1128/mBio.03103-19.1 FIG S1 IFN-λ2 and IFN-λ3/2 mRNA expression levels in HEV RNA PAMP-stimulated Tet-On RIG-I Huh7-S10-3 cells. RIG-I (a), IFN-β (b), IFN-λ1 (c), IFN-λ2 (d), and IFN-λ3/2 (e) mRNA expression levels in HEV RNA PAMP-stimulated Tet-On RIG-I cells at 18 h poststimulation were estimated using gene-specific RT-qPCR. Fold change was calculated using the 2^−ΔΔ^*^CT^* method. RPS18 was used as a housekeeping control. ^a^, *P* ≤ 0.05 versus SL3 using paired Student *t* test. Data represent means ± SEMs from biological duplicates of one experimental setup. Download FIG S1, PDF file, 0.1 MB. Copyright © 2020 Sooryanarain et al. 2020 Sooryanarain et al. This content is distributed under the terms of the Creative Commons Attribution 4.0 International license.

**FIG 5:**
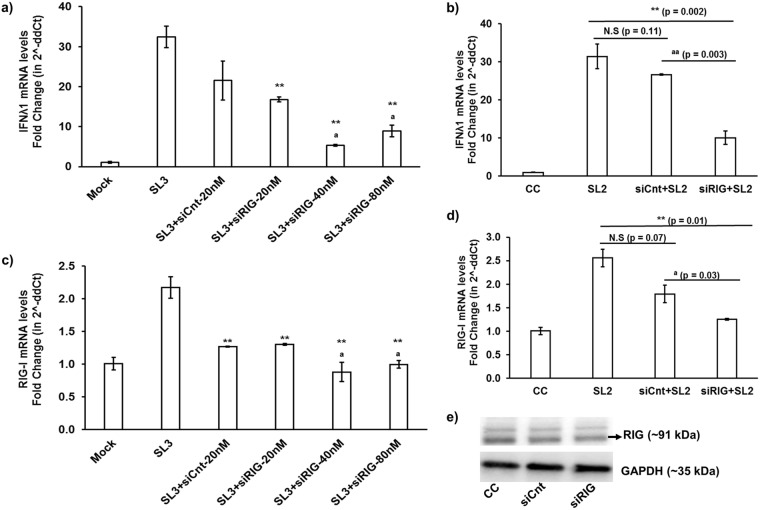
IFN-λ1 mRNA expression levels in SL3- and SL2-stimulated Huh7-S10-3 cells under RIG-I knockdown conditions. Huh7-S10-3 cells were transfected with control siRNA (siCnt) or RIG-I siRNA (siRIG). At 24 h posttransfection, the cells were transfected with SL3 (a and c) or SL2 (b and d). The mRNA expression levels of IFN-λ1 and RIG-I were estimated using gene-specific RT-qPCR. The fold change was calculated compared to unstimulated cells (mock), using the 2^−ΔΔ^*^CT^* method. RPS18 was used as a housekeeping control. (e) RIG-I protein levels in siCnt- and siRIG-transfected cells at 24 h posttransfection. **, *P* < 0.01 versus SL3/SL2-stimulated cells; ^a^, *P* < 0.05; ^aa^, *P* < 0.01 versus siCnt+SL3/SL2-stimulated cells; NS, not significant. Data represent means ± standard deviations (SDs) of results from two independent experiments.

We further determined the IFN mRNA levels in HEV RNA PAMP-stimulated Huh7-S10-3 liver cells under RIG-I knockdown conditions. Since we showed that HEV SL3 and SL2 had a stronger IFN induction potential than SL1, we therefore used HEV SL2/SL3 as the RIG-I PAMP in this particular experiment. We showed that when the RIG-I was knocked down using small interfering RNA (siRNA) in Huh7-S10-3 cells, the potential of HEV SL3 (Fig. 5a) and SL2 (Fig. 5b) to induce IFN-λ1 mRNA expression was significantly reduced compared to that of the control siRNA-treated samples.

### Enhanced interferon regulatory factor 3 nuclear localization in Huh7-S10-3 liver cells stimulated with HEV SL3.

**FIG 6:**
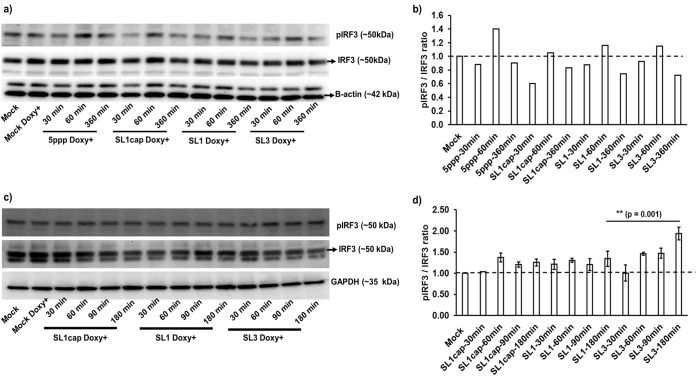
Phosphorylated IRF3 levels in HEV RNA PAMP-stimulated Tet-On RIG-I Huh7-S10-3 liver cells. Tet-On RIG-I Huh7-S10-3 cells were stimulated with various HEV RNA PAMPs (SL1cap, SL1, SL3), 5′ppp (RIG-I agonist), or left unstimulated (mock) in the presence (Doxy+) or absence of doxycycline. The cells were harvested at the indicated time points and probed for phospho-IRF3 (pIRF3) and total-IRF3 (IRF3) by Western blotting. β-Actin or GAPDH was used as a loading control. (a and c) Representative Western blots of p-IRF3 and IRF3. (b and d) Fold changes in pIRF3/IRF3 as estimated by densitometric analyses of the Western blots. Data in panel d represent means ± SDs of results from two independent experiments.

The RIG-I pathway stimulates interferon regulatory factor 3 (IRF3) activation for subsequent IFN mRNA expression (10). Therefore, in this study we quantified the levels of phosphorylated IRF3 and nuclear translocation of IRF3 to determine the activation status of IRF3 in HEV SL1cap-, SL1-, or SL3-stimulated Huh7-S10-3 cells. We compared the levels of phosphorylated IRF3 in cells 30, 60, and 360 min after transfection with 5′ppp (a commercial RIG-I agonist), SL1cap, SL1, and SL3. The results showed that PAMP stimulation led to a transient induction of IRF3 phosphorylation at 60 min posttransfection, but by 360 min, the phosphorylation levels diminished (Fig. 6a and b). Therefore, we further evaluated the levels of phosphorylated IRF3 in HEV PAMP-stimulated cells at earlier time points: 30, 60, 90, and 180 min posttransfection. We found a significantly increased phosphorylation of IRF3 in SL3-stimulated cells compared to that in the SL1-stimulated cells (Fig. 6c and d). The phosphorylated IRF3 levels in SL1cap-stimulated cells were similar to those in mock-treated cells (Fig. 6d).

**FIG 7:**
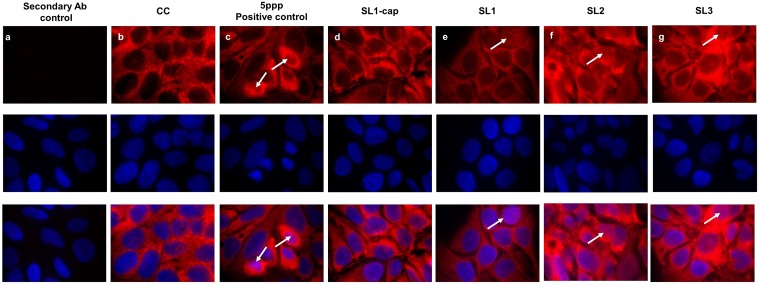
IRF3 nuclear translocation in HEV RNA PAMP-stimulated Huh7-S10-3 cells. (a) Secondary antibody control. Plain Huh7-S10-3 cells were left unstimulated (b) or stimulated with 200 ng of 5′ppp (RIG-I agonist, as a positive control) (c), SL1cap (d), SL1 (e), SL2 (f), or SL3 (g). At 18 h poststimulation, the cells were stained by IFA for IRF3 (red), and the nuclei were counterstained using DAPI (blue). Representative IRF3 nuclear localization in panels is indicated by white arrows.

Additionally, we further determined the nuclear translocation of IRF3 using immunofluorescence assay (IFA) staining of HEV RNA PAMP-stimulated cells (Fig. 7). Consistent with our Western blotting and RT-qPCR results, we observed increased IRF3 nuclear translocation in SL3-stimulated cells (Fig. 7g) compared to that in the SL1-stimulated cells (Fig. 7e), further supporting our results that HEV SL3 has a greater capability to induce IFN responses in Huh7-S10-3 liver cells than HEV SL1.

### The U-rich region is essential for the HEV UTR-induced IFN response.

**FIG 8:**
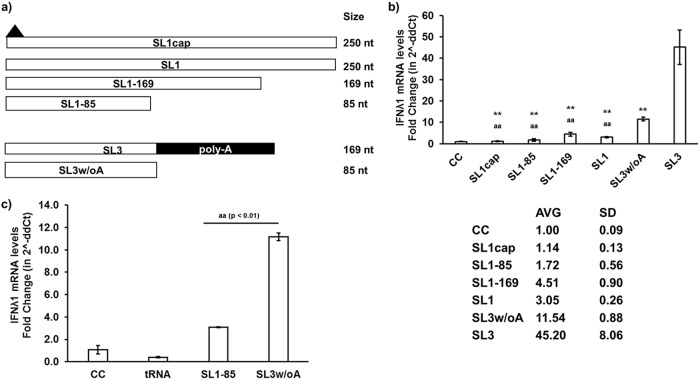
The U-rich region is essential for HEV UTR-induced IFN response. (a) Schematic representation of various lengths of SL1 and SL3 HEV RNA PAMP constructs. Capped-SL1 (SL1cap; 250 nt), SL1 (250 nt), SL1-169 (169 nt), SL1-85 (85 nt), SL3 without poly(A) tail (SL3w/oA; 85 nt), SL3 [contains poly(A) tail; 169 nt]. Black-filled triangle represents 5′ cap in SL1cap. (b and c) IFN-λ1 mRNA expression levels in HEV RNA PAMP-stimulated cells at 18 h poststimulation were estimated using gene-specific RT-qPCR. The fold change was calculated compared to unstimulated cells (CC) using the 2^−ΔΔ^*^CT^* method. RPS18 was used as a housekeeping control. **, *P* < 0.01 versus SL3-stimulated cells; ^aa^, *P* < 0.01 versus SL3w/oA-stimulated cells. Data represent means ± SDs of results from two independent experiments.

We tested various lengths of HEV SL1 and SL3 to determine the role of the U-rich region and poly(A) tail in HEV RNA PAMP-induced IFN responses. We first constructed SL1-85 (85 nucleotides [nt]), SL1-169 (169 nt), SL1 (250 nt), capped-SL1 (SL1cap; 250 nt), SL3 without poly(A) tail (SL3w/oA; 85 nt), and SL3 (contains poly(A) tail; 169 nt) (Fig. 8a). Sequence analysis revealed that the HEV P6 SL1-85 has 22.4% U, while SL1-169 has 27.2% U, and SL1 contains 27.6% U. We showed that the SL1 (85 nt) induced IFN levels similar to that of SL1cap, while SL1-169 (∼4-fold) induced IFN similarly as SL1 (∼3-fold) (Fig. 8b).

It was reported recently that the poly(A) tail is not essential for HEV RNA to induce the IFN response (11). Therefore, we also tested the potential of the SL3 without a poly(A) tail to induce an IFN response. We found that the loss of the poly(A) tail reduced the IFN activation potential of SL3 compared to that containing the poly(A) tail (Fig. 8b) but did not completely abolish the IFN-inducing capacity. To further confirm the activation capacity of HEV UTR, we used tRNA as a nonspecific inducer control in our experimental setup. As expected, we found that tRNA did not induce IFN-λ1 mRNA levels, while SL1-85 induced lower levels of IFN-λ1 mRNA than SL3 without the poly(A) tail (Fig. 8c). Therefore, the results suggest that the U-rich region is a major contributing factor for HEV UTR PAMPs, while the presence of a poly(A) tail may enhance HEV UTR-induced IFN responses.

### U-rich region variability and secondary structure stability of HEV UTRs.

**FIG 9:**
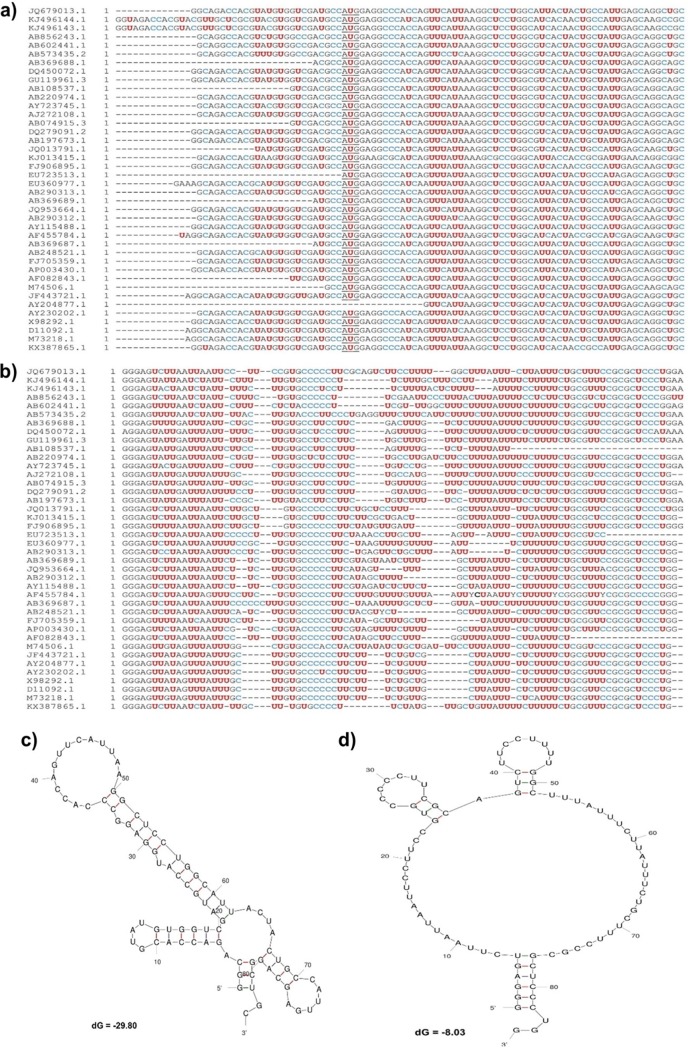
U-rich region variability and secondary structure stability of HEV UTRs. Genetic variability in the U-rich regions among genotypes 1 to 8 HEV genomic sequences was determined by a multiple-sequence alignment of SL1-85 (85 nt) (a) and SL3 without poly(A) tail (SL3w/oA; 85 nt) (b) using the MEGA6 software program (36). The U residues are marked in red, and the C residues are marked in blue. The initiation codon AUG of HEV ORF1 in SL1-85 is underlined. The secondary structures of SL1-85 (c) and SL3w/oA (d) were predicted using the mFOLD server.

We examined the genetic variability of the U-rich region in genotypes 1 to 8 HEV genomic sequences containing the full coverage of the SL1-85 (85 nt) and SL3 without poly(A) tail (SL3w/oA; 85 nt) against the genotype 3 HEV Kernow P6 sequence. We found that the SL3w/oA had a higher U content (average, 46.6%; range, 41.4% to 56.6%) than the SL1-85 (average, 22.4%; range, 17.9% to 28.9%). Our analysis also revealed that the U-rich region is mostly seen in the 3′ UTR among different HEV genotypes, and the poly(A) tail varies from strain to strain within a genotype (Fig. 9a and b). Therefore, we speculate that the observed variation in PAMP activity of the HEV UTR may be attributed to the genetic variation in the U-rich regions. Furthermore, we also determined the secondary structure of SL1-85 and SL3w/oA using the mFOLD server (22). The *in silico* secondary structure prediction showed that the SL1-85 secondary structure has a more stable thermodynamic minimum free energy (Δ*G*, −29.80), while the SL3w/oA has a thermodynamically less stable minimum free energy (Δ*G*, −8.03) and therefore a more flexible secondary structure (Fig. 9c and d).

### The 3′ UTR of HEV induced predominantly type I IFN responses in enterocytes.

**FIG 10:**
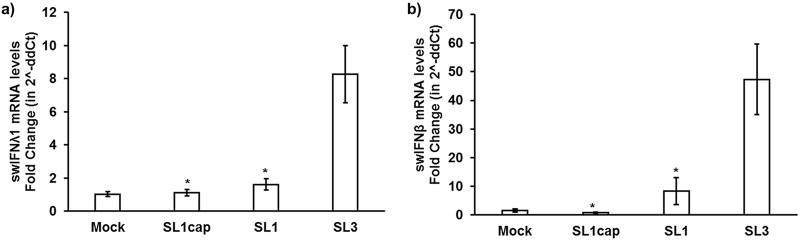
The HEV 3′ UTR induced predominantly type I IFN responses in enterocytes IPEC-J2 cells. Swine type III IFN (swIFN-λ1) (a) and type I IFN (swIFN-β) (b) mRNA expression levels in HEV RNA PAMP-stimulated IPEC-J2 cells at 18 h poststimulation were estimated using gene-specific RT-qPCR. Fold change was calculated using the 2^−ΔΔ^*^CT^* method. Swine RPS18 was used as a housekeeping control. *, *P* ≤ 0.05 versus SL3 using paired Student *t* test. Data represent means ± SEMs from two independent experiments.

A tissue-specific IFN response has been reported during HEV infection (23, 24). Therefore, in addition to liver tissues from HEV-infected pigs and Huh7-S10-3 liver cells, we also determined the IFN mRNA expression levels in HEV SL1- and SL3-stimulated swine enterocyte IPEC-J2 cells using gene-specific RT-qPCR. The IPEC-J2 cells were used in this study since they mimic human enterocytes physiologically and this cell line is neither transformed nor tumorigenic in nature (25). Most importantly, HEV replication has been detected in small intestine tissues of HEV-infected animals (18) as well as in IPEC-J2 cells (data not shown). As observed with Huh7-S10-3 liver cells in this study, we found that HEV SL3 also induced higher levels of IFN mRNA expression in IPEC-J2 cells than in the SL1-stimulated cells (Fig. 10a and b). However, unlike Huh7-S10-3 liver cells, the type I IFN mRNA expression level was significantly higher (∼40-fold) than the type III IFN mRNA expression level (∼8-fold) in HEV SL3-stimulated IPEC-J2 cells. The results suggest that a differential type III and type I IFN response induced by HEV RNA PAMPs depends on viral RNA motifs and is apparently also host tissue specific.

## DISCUSSION

HEV UTRs comprise a 5′ UTR, a *cis*-reactive region at the 3′ UTR, and a stem-loop junction region between the end of ORF1 and start of ORF2/3 (14). The *cis*-reactive region of the 3′ UTR is critical for viral replication, and the stem-loop junction region acts as a promoter for HEV subgenomic RNA expression (15–17). In addition to playing an important role in viral replication, the UTRs of viral RNAs are also recognized by host PRRs such as RIG-I. The RIG-I-mediated innate immune response via IFN is known to play an important role in HEV infection (11, 12, 20). However, the HEV RNA PAMPs which are recognized by RIG-I are currently unknown.

In this study, we demonstrated that the HEV RNA PAMPs with U-rich regions in the UTRs (SL3 and SL2) induced higher levels of IFN responses and that the presence of poly(A) further enhanced the IFN induction potential. We showed that the HEV 5′ UTR (SL1) induced IFN responses at lower levels than the HEV 3′ UTR (SL3) or the stem-loop junction region (SL2). RIG-I preferentially binds to viral RNA PAMPs of <300 bp (21), and it is known that the sequence composition and presence of cap or free phosphate group at the 5′ end influence the RIG-I recognition (9). It was also reported that the hepatitis C virus (HCV) 3′ UTR with a poly(U/UC) region was a strong RIG-I agonist (26). In this study, we demonstrated that the U-rich region in HEV 3′ UTR had a higher IFN-inducing potential than the 5′ UTR. Analyses of genotypes 1 to 8 HEV genomic sequences further confirmed that the 3′ UTR had U-rich regions, with 46.6% U content on average compared to the 5′ UTR with an average of 22.4% U content. We further demonstrated that the loss of the poly(A) tail in HEV SL3 significantly reduced the IFN induction potential of SL3, although it did not completely abolish its IFN induction potential. This was in corroboration with a previous study which demonstrated the IFN induction potential by HEV full-length genomic RNA was independent of the poly(A) tail (11). A poly(A) sequence of 50 nucleotides or more in length is known to induce an IFN response via RIG-I (26). The HEV P6 genome has a poly(A) tail of 84 nucleotides in length, although the length of poly(A) tails varies among HEV genotypes. Analysis of the prototypic genotypes 1 to 8 HEV strains revealed that the length of poly(A) tails varied among different HEV strains, in the range between 4 to 35 nucleotides (data not shown). Therefore, taken together, the results suggest that the observed variability in the IFN induction potential of different HEV RNA PAMPs apparently depends more on viral RNA motif sequence composition, i.e., presence of a U-rich region, while the presence of longer poly(A) tails may enhance IFN induction.

Recent studies showed that HEV induced IFN in both host cell-dependent and genotype-specific manners (23, 24). Therefore, in this study, we tested the IFN induction ability by HEV RNA PAMPs in both hepatocytes and enterocytes, since the primary site of HEV replication is the small intestine before reaching the target organ (the liver). Our results showed that the HEV RNA PAMP SL3 had a higher IFN induction potential than SL1. Importantly, the HEV RNA PAMPs predominantly induced a type I IFN response in swine enterocytes, while it induced predominantly type III IFN responses in human hepatocytes and swine liver tissues. Therefore, our results indicated that the HEV RNA PAMP-induced IFN response was also dependent on host target cell type in addition to the PAMP sequence composition. Furthermore, our *in vitro* data obtained in human hepatocytes corroborated our *in vivo* data obtained from liver tissue samples of HEV-infected gnotobiotic and conventional pigs.

Studies have also shown that HEV predominantly induces type III IFN in hepatocytes (20), and high serum IFN-λ3 levels has been observed in patients with acute HEV infection (27). Various IFN subtypes contribute to the antiviral effect against HEV (27, 28), and HEV is known to regulate the IFN response dependent on both host and viral factors (23, 24, 29). Therefore, it is possible that type III IFN may be the predominant IFN to mediate antiviral effects in the liver, while type I IFN may play an important role in enterocytes, the initial site of HEV replication. Interestingly, type III IFN is known to contribute to antiviral responses at the intestinal mucosal surface (30, 31); however, in this study, we found that HEV RNA PAMPs induced higher type I IFN mRNA levels than type III IFN levels in enterocytes. Therefore, more in-depth *in vivo* studies are warranted to more definitively delineate the role of type I and type III IFNs at various target organs during HEV infection. Such an in-depth *in vivo* study in the future would be of vital importance to understand the immunopathogenesis of HEV infection, since it has been shown that type I and type III IFNs tend to induce distinct antiviral responses during rotavirus infection in a mouse model (32).

In conclusion, we demonstrated in this study that the U-rich regions in the HEV UTRs act as stronger RIG-I viral RNA PAMPs. We also revealed that HEV UTR PAMPs induce type I and type III IFN responses in a cell type-dependent fashion. Studies have shown that various IFN subtypes exert different spatial and temporal activation kinetic patterns *in vivo* (33) and also use distinct mechanisms to establish antiviral states *in vitro* (34). Therefore, the complexity of virus-host interactions modulates the expression of various IFN subtypes and, in turn, controls the antiviral response kinetics at a given target organ.

## MATERIALS AND METHODS

### Cells, virus, and other reagents.

293T cells, Huh7-S10-3 liver cells, and IPEC-J2 intestinal epithelial cells were maintained in Dulbecco’s modified Eagle medium (DMEM) containing 10% fetal calf serum (FCS), 1× antibacterial-antimycotic, and 1× minimal essential amino acid (Gibco-Thermo Fisher, MA, USA). The HEV genotype 3 Kernow P6 virus stock was prepared by transfecting Huh7-S10-3 cells with *in vitro*-transcribed capped RNA transcripts from the HEV Kernow P6 infectious clone. The following antibodies and siRNA were used in this study: anti-IRF3 (1:1,000; SCBT, CA, USA), anti-phospho-IRF3 (1:1,000; Millipore, MA, USA), anti-glyceraldehyde-3-phosphate dehydrogenase-horseradish peroxidase (anti-GAPDH-HRP) (1:5,000; Invitrogen, MA, USA), bovine-anti-rabbit-HRP (1:7,000; SCBT, CA, USA), donkey-anti-rabbit-rhodamine red Fab (1:2,000; Jackson Laboratory, ME, USA), and siRIG-I (20 μM; SCBT, CA, USA). The IFN-β-firefly luciferase plasmid and TK-*Renilla* luciferase plasmid for the IFN promoter assay were purchased from Promega (WI, USA). The pLIX-402 lentiviral vector and second-generation lentiviral packing vectors (psPAX2 and pMD2.G) were procured from Addgene (MA, USA).

### Liver tissues from conventional and gnotobiotic pigs experimentally infected with a genotype 3 human HEV.

In previous unrelated studies, we experimentally infected conventional pigs (18) and gnotobiotic pigs (19) with a genotype 3 strain of human HEV. Samples of liver tissues from infected and control pigs were collected during necropsy and stored at −80°C. The convenient liver tissues collected from HEV-infected conventional pigs and gnotobiotic pigs at 4 weeks postinfection (wpi) were used in the present study to determine the differential type I and type III IFN responses in HEV-infected animals.

### Cloning and generation of RIG-I-containing lentivirus.

The full-length human RIG-I coding DNA sequence (CDS) was cloned into pLIX-402 lentivirus vector containing a doxycycline-inducible promoter. The lentivirus particles containing human RIG-I (lentivirus-hRIG) were generated using 293T cells as per an established protocol (35). Briefly, 293T cells were transfected with pLIX402-hRIG-I and second-generation lentivirus packaging vectors (psPAX2 and pMD2.G). The transfected cells were maintained in DMEM plus 10% FCS at 37°C and 5% CO_2_. At 48 h posttransfection, the cell culture supernatant containing lentivirus-hRIG was collected and clarified using low-speed centrifugation (2,000 × *g*, for 10 min). The clarified lentivirus-hRIG preparation was then aliquoted and stored at −80°C until use.

### *In vitro* transcription of HEV UTRs.

The HEV RNA PAMPs were produced from T7 promoter-linked HEV UTR PCR products. Several T7 promoter linked HEV UTR fragments spanning 1 to 250 nt (stem-loop 1 [SL1], 250 nt), nt 5250 to 5414 (SL2; 165 nt), and nt 7331 to 7499 [SL3; 169 nt, contains poly(A) tail] were generated by PCR with specific primers using the genotype 3 HEV Kernow P6 infection cDNA clone as the template. The purified T7 promoter-inked PCR products were subsequently used to generate HEV RNA PAMPs, using the T7 *in vitro* transcription kit (NEB, MA, USA).

### Real-time RT-qPCR.

Total cellular RNAs were isolated using TRI-Reagent (MRC, OH, USA) as per the manufacturer’s protocol. The cDNA was synthesized using random primers with a cDNA kit (ABI-Thermo Fisher, MA, USA). The mRNA levels of IFN-λ1/3 and IFN-β/α were estimated using gene-specific primers (Table 1) using the Sybr green qPCR kit (ABI-Thermo Fisher, MA, USA). The qPCR conditions were as follows: 95°C for 2 min, followed by 40 cycles of 95°C for 5 s, 60°C for 10 s, and 72°C for 20 s.

**TABLE 1:** Oligonucleotide primers used in this study

ID^*a*^	Sequence (5′→3′)	Purpose
T7pro+SL1-250-FP^*b*^	TCCTGTAATACGACTCACTATAGGCAGACCACGTATG	SL1 RNA PAMP PCR
SL1-250 RP	AGTTCATTGTGTATGACCCGCTGG	SL1 RNA PAMP PCR
SL1-169RP	TTGATAAGAATCTCAGTTTGTAGACGGGAT	SL1 RNA PAMP PCR
SL1-85RP	GCAGCCTGCTCAATGGCAGTAGTAA	SL1 RNA PAMP PCR
T7pro+Nt5250-5414 FP^*b*^	TCCTGTAATACGACTCACTATAGGCAAGGCCCACTTTACAGAGAC	SL2 RNA PAMP PCR
Nt5250-5414 RP	GCGGGCAGCATAGGCAGAAAC	SL2 RNA PAMP PCR
T7pro+Nt7331-7499 FP^*b*^	TCCTGTAATACGACTCACTATAGGGAGTCTTAATTAATTCCTTCCGTGCCC	SL3 RNA PAMP PCR
Nt7331-7499 RP	poly(T) (84 bases)	SL3 RNA PAMP PCR
Nt7331-7415RP	CCAGGGAGCGCGGAAAGCAGAAATAAG	SL3 RNA PAMP PCR
swIFNβ-FP	TGTGGAACTTGATGGGCAGA	qPCR
swIFNβ-RP	GGCACAGCTTCTGTACTCCTT	qPCR
swIFNα-FP	CAGTTCTGCACTGGACTGGA	qPCR
swIFNα-RP	CACAGGGGCTGTAGCTCTTC	qPCR
swRPL32-FP	CTCAGACCCCTTGTGAAGCC	qPCR
swRPL32-RP	TCTGGCCCTTGAACCTTCTC	qPCR
swRPS18-FP	CATCGACCTCACCAAGAGGG	qPCR
swRPS18-RP	CCTGGCTGTACTTCCCATCC	qPCR
swIFNλ1-FP	ACGTCGAACTTCAGGCTTGC	qPCR
swIFNλ1-RP	GGCAGCCTTGGGACTCTTTC	qPCR
swIFNλ3-FP	CCTCTTGGAGGACTGGAACTG	qPCR
swIFNλ3-RP	CTGTGCAGGGATGAGTTCGC	qPCR
hIFNλ1-FP	AAAAAGGAGTCCGCTGGCTG	qPCR
hIFNλ1-RP	TCAGACACAGGTTCCCATCG	qPCR
hRIG-I – FP	AGAGCACTTGTGGACGCTTT	qPCR
hRIG-I – RP	ATACACTTCTGTGCCGGGAG	qPCR
hRPS18-FP	TGATCCCTGAAAAGTTCCAGCA	qPCR
hRPS18-RP	CTTCGGCCCACACCCTTAAT	qPCR
hIFNβ-FP	AGTGTCAGAAGCTCCTGTGGC	qPCR
hIFNβ-RP	TGAGGCAGTATTCAAGCCTCC	qPCR

a ID, identifier.

b T7 promoter sequence is underlined.

### Western blot analyses.

The Huh7-S10-3 liver cells were stimulated with different HEV RNA PAMPs for various durations. The cells were transfected with 200 ng of various HEV RNA PAMPs using Lipofectamine 2000 (Invitrogen-Thermo Fisher, MA, USA). The stimulated cells were lysed at different time points using RIPA buffer containing 1× protease/phosphatase inhibitor. The cell lysates were clarified by spinning at 14,000 rpm for 5 min at 4°C. A total of 60 μg of cell lysate was loaded into each lane and resolved using 4% to 20% gradient SDS-PAGE gel. The proteins were subsequently transferred onto a polyvinylidene difluoride (PVDF) membrane and blocked for 1 h using 5% bovine serum albumin (BSA) in phosphate-buffered saline containing 0.1% Tween 20 (PBST). The membranes were then probed using rabbit-phospho-IRF3 (1:1,000 dilution) and rabbit-IRF3 (1:1,000 dilution). Bovine-anti-rabbit-HRP (1:7,000 dilution) was used as the secondary antibody. GAPDH was used as a loading control and was detected using anti-GAPDH-HRP (1:5,000 dilution). The blot was developed using ECL reagent (SCBT, CA, USA) and imaged with a Bio-Rad imagining system. ImageJ software (NIH, MD, USA) was used for densitometric analysis of the Western blot.

### Immunofluorescence assay.

Huh7-S10-3 cells were seeded onto 8-chamber slides and transfected with various HEV-RNA PAMPs, RIG-agonist 5′ppp (Invivogen, CA, USA) as a positive control, or left unstimulated (mock control). At 18 h poststimulation, the cells were fixed using ice-cold acetone. The cells were then blocked using 10% goat serum in PBST and probed for IRF3 using rabbit-anti-IRF3 antibody (1:150 dilution in blocking buffer). Donkey-anti-rabbit-rhodamine red Fab (1:2,000 dilution in blocking buffer) was used as the secondary antibody. DAPI (4′,6-diamidino-2-phenylindole; 1:10,000 dilution) was used to counterstain the nuclei.

### Dual luciferase assay.

In a 96-well plate, Huh7-S10-3 cells were transfected with 100 ng of IFN-β-firefly luciferase promoter plasmid (reporter plasmid) and 5 ng TK-*Renilla* luciferase plasmid (transfection control plasmid) per well using Lipofectamine 2000 (Invitrogen-Thermo Fisher, MA, USA) reagent as per the manufacturer’s protocol. At 18 h posttransfection, the cells were transfected with 100 ng of different HEV RNA PAMPs. At 12 h post-HEV RNA PAMP stimulation, the cells were lysed and the levels of luciferase activities were measured using a Dual-Glo luciferase assay kit (Promega, WI, USA).
