# Supplementary figures and images for: The U-Rich Untranslated Region of the Hepatitis E Virus Induces Differential Type I and Type III Interferon Responses in a Host Cell-Dependent Manner
Source: mBio. 2020 Jan 14;11(1):e03103-19. doi: 10.1128/mBio.03103-19 (PMC6960293; doi:10.1128/mBio.03103-19)

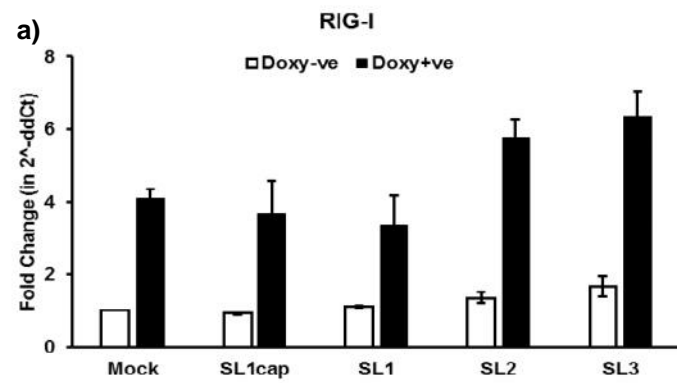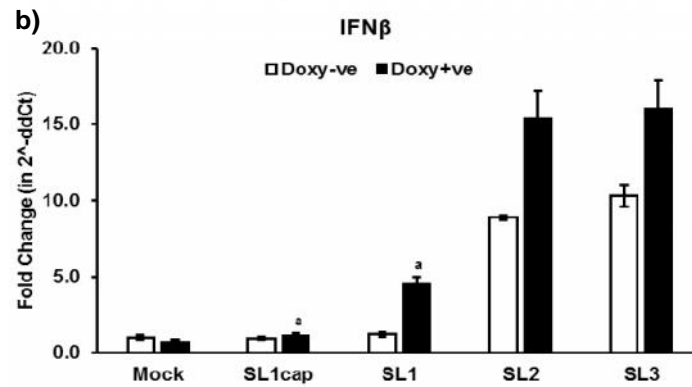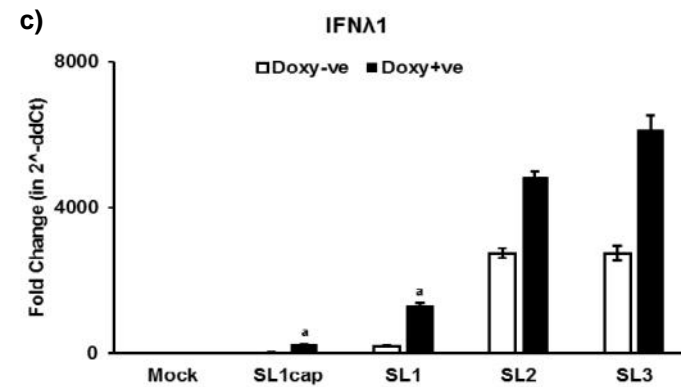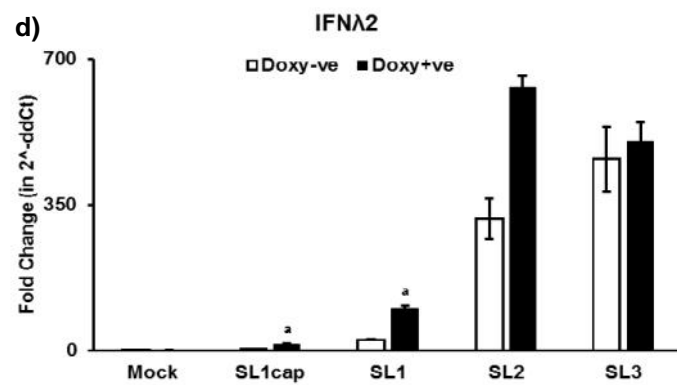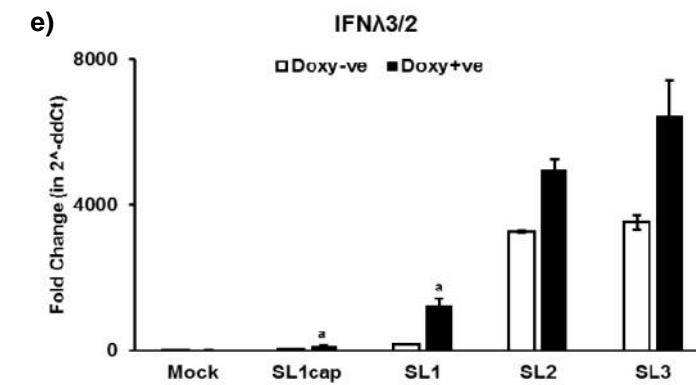

Supplement: FIG S1 [file mBio.03103-19-sf001.pdf]
